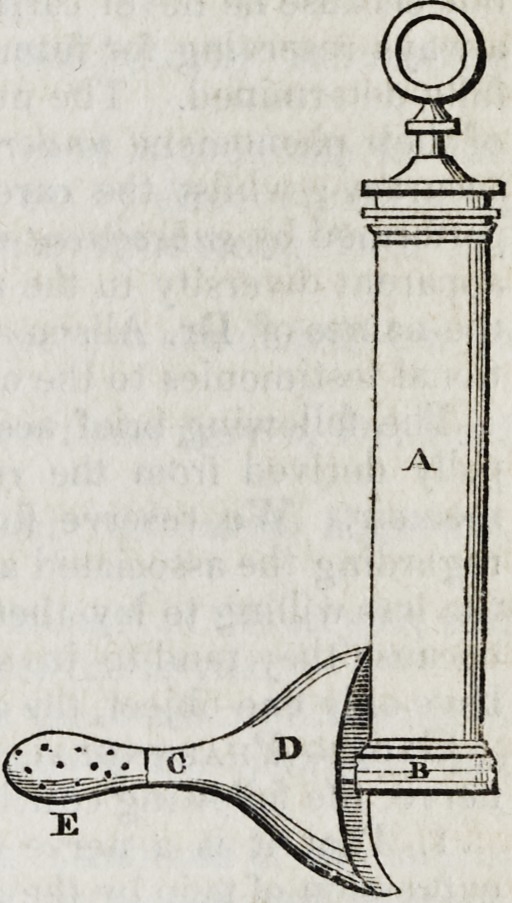# Improved Female Syringe

**Published:** 1838-01

**Authors:** Heber Chase


### IMPROVED FEMALE SYRINGE. BY HEBER CHASE, M.D.

THE superiority which this instrument possesses consists in its perfect adaptation to the anatomical form of the external organs of generation.

A, the cylinder, is about five inches in length, with a caliber of one inch. Projecting from its lower extremity, B, at an angle of about eighty-five degrees, is a tube of one inch and a half in length and six lines in diameter, and terminating at C by a male screw in the shield D, now to be described. The shield is of a conoid form, produced considerably near the truncated summit, and laterally compressed; about four inches in length, half an inch in diameter at the apex, and has about three inches vertical and two inches transverse diameter at its base. The superior extremity of the vertical diameter rests against the cylinder of the instrument, while the inferior extremity is carried backwards and downwards so as to press on the perineum a few lines posteriorly. Upon the extremity of this shield is placed an ivory tube, E, extending one-third of its whole length. This tube, from its connexion with the shield at C, is gradually increased towards its extremity, and terminates in a diameter of ten lines, where it is perforated by from twelve to fifteen holes all around its bulbous extremity. The ivory tube may be removed from the shield at C, where it is attached by means of a screw. The shield itself may be removed from the instrument in the same manner, and at nearly the same point.

*Directions for Use.*—The bulbous extremity of the instrument should be introduced into the vagina, and carried, backwards and upwards, nearly or quite to the os uteri, the base of the shield closing the vagina at its orifice. When the contents of the syringe are thrown into the vagina, the fluid, of whatsoever nature, is projected not only against and around the os uteri, but cleanses also, by means of the numerous orifices in the bulb, the other parts of the canal, while the shield prevents its escape.

The syringe is made of the usual material, pewter, with the exception of the bulbous extremity of the shield which, as before stated, is of ivory. In manufacturing the instrument, care should be taken that the shield be highly finished, and the holes in the bulbous extremity be made smooth, so that no friction upon the internal parts may follow its use. Ivory is far preferable to bone or other substances for forming the bulb, from the facility with which it may be polished.

*American Journal of Medical Sciences. February,* 1837.

## Figures and Tables

**Figure f1:**